# Beyond blood pressure: the renin-angiotensin system as an innovative driver and therapeutic target in pathological scarring

**DOI:** 10.3389/fphar.2026.1792119

**Published:** 2026-03-05

**Authors:** Bang-Hui Shi, Xin-Ge Zhang, Qing-Qing Fang, Kai Xu, Xiao-Ling Chen, Wei-Qiang Tan, Shou-Jie Wang

**Affiliations:** 1 Department of Plastic Surgery, The Fourth Affiliated Hospital of School of Medicine, and International School of Medicine, International Institutes of Medicine, Zhejiang University, Yiwu, Zhejiang, China; 2 Department of Plastic Surgery, Sir Run Run Shaw Hospital, Zhejiang University School of Medicine, Hangzhou, Zhejiang, China; 3 Department of Plastic Surgery, The First Affiliated Hospital, School of Medicine, Zhejiang University, Hangzhou, Zhejiang, China

**Keywords:** angiotensin-converting enzyme, clinical translation, fibrosis, hypertrophic scar, keloid, pathological scarring, renin–angiotensin system, targeted therapy

## Abstract

Pathological scarring, a fibroproliferative disorder, imposes a substantial burden on affected individuals. This review explores the pivotal role of the local cutaneous renin-angiotensin system (RAS) in the pathogenesis of pathological scarring. We summarize evidence demonstrating how the pro-fibrotic angiotensin II/angiotensin II type 1 receptor (Ang II/AT1R) axis drives scar formation by promoting fibroblast proliferation, inflammation, and excessive extracellular matrix (ECM) deposition. Concurrently, we examine the interactions between RAS and other fibrotic pathways, as well as inflammation and reactive oxygen species (ROS). Importantly, the review highlights the significant therapeutic potential of targeting this pathway with RAS inhibitors—specifically angiotensin-converting enzyme inhibitors (ACEIs) and angiotensin receptor blockers (ARBs)—particularly in topical formulations. We also outline recent advances in next-generation RAS therapies. Finally, we summarize current limitations and challenges in clinical translation, emphasizing the need for advanced clinical trials and precision medicine strategies to facilitate its clinical adoption.

## 1 Introduction

The condition of pathological scarring, which includes the development of hypertrophic scars and keloids, is characterized by aberrant wound healing processes and can impose significant aesthetic, functional, and psychosocial burdens on affected individuals (Robles and Berg, 2007; Gao et al., 2013; Li et al., 2024). These burdens may include pruritus, pain, contracture, and visible disfigurement, which may lead to impaired mobility and potential mental health issues (Trace et al., 2016). Despite the high global incidence of pathological scars following surgery or trauma, effective preventive and treatment options remain limited (Murakami and Shigeki, 2024; Lee and Jang, 2018).

From a pathophysiological standpoint, pathological scars are characterized by chronic inflammation and excessive fibrosis. Persistent activation of fibroblasts and myofibroblasts has been shown to trigger imbalanced extracellular matrix (ECM) deposition and irregular collagen architecture, resulting in thickened, rigid scars (Huang and Ogawa, 2020). Previous research has identified factors such as transforming growth factor-β (TGF-β), sustained inflammatory signaling, and mechanical stress as being central to scar pathogenesis (Xia et al., 2021; Xing et al., 2024). TGF-β is particularly well recognized for its roles in promoting fibroblast proliferation, ECM accumulation, and myofibroblast conversion (Zhang et al., 2020; Tan et al., 2023). Nevertheless, therapeutic interventions targeting the TGF-β axis have so far yielded inconsistent clinical outcomes, and recurrence rates remain high, highlighting the urgent need for novel mechanistic insights and treatment strategies (Du et al., 2024).

In recent years, emerging evidence has identified the renin–angiotensin system (RAS), which was traditionally established as a systemic regulator of hemodynamics, as a critical contributor to fibrosis and scar development (Yang et al., 2019; Bernasconi and Nyström, 2018; AlQudah et al., 2020). Recent studies have demonstrated that components of local skin RAS, including angiotensin II (Ang II) and the angiotensin II type 2 receptor (AT1R), are overexpressed in pathological scars and drive profibrotic processes through enhanced inflammation, angiogenesis, and fibroblast activation, whereas angiotensin II type 2 receptor (AT2R) signaling exerts counter-regulatory, antifibrotic effects (Maranduca et al., 2023; Greif et al., 2023).

Furthermore, innovative therapeutic modalities inspired by these insights have demonstrated considerable potential. For instance, losartan-loaded microneedle patches have been developed to deliver angiotensin receptor blockers more effectively into scar tissue, significantly reducing hypertrophic scar formation in animal models via enhanced transdermal delivery (Huang Y. et al., 2023). Concurrently, compound losartan creams incorporating chitosan and asiaticoside have been shown to inhibit scarring in murine models by blocking the TGF-β/Smad pathway (Zhao et al., 2022). These innovative delivery systems overcome limitations of traditional topical applications and hold translational potential for future scar management.

The following conclusions are drawn from the findings presented above, together with our team’s original contributions, which include the discovery of the dual origin of angiotensin-converting enzyme (ACE) in both bone marrow-derived and skin-resident sources, and the elucidation of the manner in which ACE inhibitors concurrently suppress both the canonical (TGF-β/Smad (suppressor of mothers against decapentaplegic)) and noncanonical Transforming Growth Factor-β-Activated Kinase 1 (TAK1) fibrotic pathways (Fang et al., 2018a). This review synthesizes the available mechanistic and therapeutic evidence of RAS in scar formation, and expands the discussion to include crosstalk with other key fibrotic pathways and innovative biomaterial strategies. Finally, a critical evaluation of the limitations of current knowledge is required to facilitate optimal clinical translation.

## 2 The renin–angiotensin system beyond circulation

### 2.1 Classical systemic RAS

The RAS has historically been recognized as a circulating hormonal cascade that regulates blood pressure, electrolyte balance, and vascular tone (Bader et al., 2024; Jia and Sowers, 2021). The process of hepatic angiotensinogen cleavage is initiated by renin, resulting in the generation of angiotensin I (Ang I). This is subsequently converted by ACE into the bioactive peptide Ang II (Belova, 2000). Ang II has been demonstrated to mediate its biological effects primarily through two receptor subtypes: the AT1R and AT2R (Chaudhary, 2021).

AT1R signaling has been demonstrated to be strongly profibrotic and proinflammatory (Yoshiji et al., 2007). It has been demonstrated that this process stimulates fibroblast proliferation, promotes ECM deposition, and induces inflammatory cytokine release via TGF-β/Smad, mitogen-activated protein kinase (MAPK), and nuclear factor kappa-light-chain-enhancer of activated B cells (NF-κB) pathways (Greif et al., 2023; Forrester et al., 2018a). In contrast, AT2R activation generally counteracts AT1R effects, exerting antifibrotic, anti-inflammatory, and vasodilatory properties (Karnik et al., 2015). This dualistic signaling underscores the complexity of RAS regulation beyond hemodynamics and suggests potential therapeutic opportunities in fibrotic diseases.

### 2.2 Local RAS in the skin

In addition to its systemic role, RAS is increasingly recognized as a locally active system within multiple tissues, including the skin (Tan et al., 2018). Local RAS functions through autocrine, paracrine, and intracrine mechanisms that are distinct from circulating RAS. These mechanisms are characterized by the capacity of keratinocytes, fibroblasts, and immune cells to express RAS components (Maranduca et al., 2023; Hedayatyanfard et al., 2020).

Recent studies have demonstrated that the expression of Ang II, ACE, AT1R, and AT2R is significantly increased in pathological scar tissue in comparison to normal skin (Zhao et al., 2022; Hu et al., 2020). In hypertrophic scars and keloids, Ang II/AT1R signaling has been shown to drive fibroblast activation, promote collagen I and III synthesis, and sustain chronic inflammation, whereas AT2R expression is relatively reduced, thereby promoting fibrosis (Zhao et al., 2022). Furthermore, it has been demonstrated that ACE can be derived from both bone marrow-derived inflammatory cells and skin-resident cells, which further amplifies local Ang II production and scar progression (Fang et al., 2018a).

Collectively, these findings lend support to the concept that local cutaneous RAS plays an integral role in wound repair, inflammation, and fibrogenesis, thus establishing it as a promising target for therapeutic intervention in pathological scarring.

## 3 RAS as a driver of pathological scarring

### 3.1 Fibrotic signaling pathways

**FIGURE 1:**
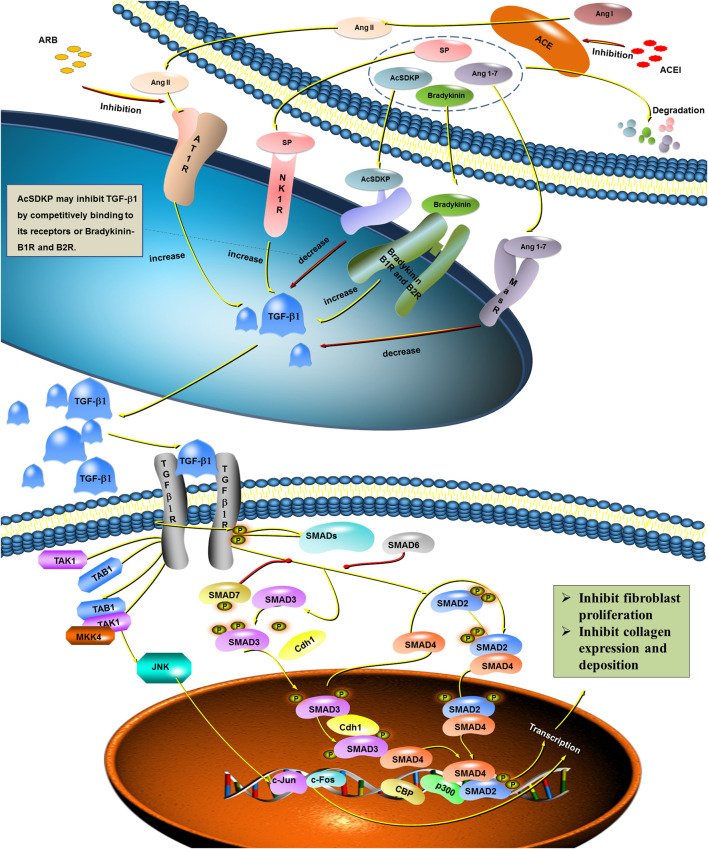
AngII–AT1R signaling and other potential ways that influence fibrotic remodeling in pathological scars.

Ang II/AT1R signaling has been identified as a central driver of fibrotic remodeling in pathological scars (Figure 1). A pivotal mechanism in this process is the activation of the canonical TGF-β/Smad pathway. The binding of Ang II to AT1R has been demonstrated to promote the generation of reactive oxygen species (ROS), which in turn activate latent TGF-β1 (Maranduca et al., 2023; Forrester et al., 2018b). The activation of TGF-β1 has been shown to phosphorylate Smad2/3, thereby enabling the nuclear translocation of Smad complexes and the transcription of profibrotic genes such as collagen type I/III and fibronectin. This process ultimately leads to the enhancement of ECM accumulation and scar hypertrophy (Greif et al., 2023; Zhao et al., 2022).

Beyond canonical signaling, Ang II/AT1R also stimulates noncanonical cascades, particularly the TAK1 pathway. TAK1 activation subsequently stimulates downstream MAPK family members (extracellular signal-regulated kinase (ERK), c-Jun N-terminal kinase (JNK), and p38 MAP kinase (p38)) and NF-κB, thereby amplifying fibroblast proliferation and the release of inflammatory cytokines (Forrester et al., 2018a; Fang et al., 2018b; Aashaq et al., 2019). These noncanonical pathways act synergistically with Smad signaling, thereby potentiating the profibrotic phenotype of fibroblasts.

Furthermore, in the study of fibrosis mechanisms in organs such as the liver, heart, and kidneys, may also provide valuable insights for understanding the RAS network in cutaneous scarring. For instance, Ang II generated by ACE is recognized as a potent profibrotic factor, whereas angiotensin-(1–7) (Ang-(1–7)) exerts protective effects via the AT2R (Gupta et al., 2021; Molteni et al., 2003; Skultetyova et al., 2007). In addition, molecules such as substance P (SP), bradykinin, and N-acetyl-seryl-aspartyl-lysyl-proline (AcSDKP) have been shown to modulate fibroblast activity or inflammatory responses, further expanding the RAS-associated fibrotic regulatory network (Rhaleb et al., 2001; Sugioka et al., 2024). How these pathways interact with classical Ang II/AT1R signaling to collectively promote scar formation warrants further in-depth investigation.

Legend: ACE catalyzes the conversion of Ang I to Ang II, which binds to AT1R and initiates multiple profibrotic cascades. Canonical TGF-β/Smad pathway: Ang II/AT1R signaling induces ROS production, activating latent TGF-β1. Activated TGF-β1 phosphorylates Smad2/3, which forms a complex with Smad4 and translocases to the nucleus (Maranduca et al., 2023; Forrester et al., 2018b). Noncanonical TAK1 pathway: Ang II/AT1R activates TAK1, leading to downstream MAPK (ERK, JNK, p38) and NF-κB signaling. JNK phosphorylation initiates AP-1 (c-Jun/c-Fos) complex formation. Nuclear Smad complexes converge with AP-1 at target gene promoters, where recruitment of transcriptional coactivators P300/CBP induces chromatin remodeling via histone acetylation, synergistically driving transcription of profibrotic genes including α-smooth muscle actin (α-SMA), collagen type I alpha one chain (COL1A1), and collagen type III alpha one chain (COL3A1). This cascade promotes fibroblast-to-myofibroblast differentiation and excessive ECM deposition (Tan et al., 2018; Fang et al., 2018b; Aashaq et al., 2019). Associated fibrotic mediators identified in other organs: Additional molecules implicated in fibrosis—including substance P (SP, promoting fibroblast activation and collagen synthesis), bradykinin (exacerbating inflammation via endothelial stimulation), Ang-(1–7) (attenuating fibrosis through AT2R), and AcSDKP (inhibiting fibroblast proliferation and collagen synthesis)—may also contribute to the cutaneous RAS fibrotic network, though their specific roles in scar formation require further investigation.

Furthermore, Ang II/AT1R signaling has been demonstrated to disrupt the balance between matrix metalloproteinases (MMPs) and their endogenous inhibitors (TIMPs). Upregulation of TIMP-1 coupled with suppression of MMP-1 and MMP-3 decreases ECM degradation capacity, leading to excessive collagen accumulation and a disorganized scar matrix (Maranduca et al., 2023).

Collectively, the activation of both canonical and noncanonical fibrotic pathways by Ang II positions local RAS as a pivotal profibrotic axis in scar pathogenesis. Consequently, the utilization of pathway inhibitors, such as ACEIs and angiotensin receptor blockers (ARBs), may represent an efficacious approach to the inhibition of hypertrophic scarring.

### 3.2 Interaction with other key fibrotic pathways

Beyond the canonical TGF-β/Smad and non-canonical TAK1 pathways, RAS signaling intersects with several other critical fibrotic cascades, contributing to a complex regulatory network in scar formation (Kim and Kim, 2024). The protein kinase B(Akt) pathway (Wang et al., 2022), a central node in cell survival, proliferation, and protein synthesis, can be activated by Ang II via AT1R, further promoting fibroblast activation and collagen production (Mehta and Griendling, 2007). This intersection suggests that combined inhibition of RAS and Akt signaling might yield synergistic antifibrotic effects.

The Wnt/β-catenin pathway, crucial in development and fibrosis, also shows interaction with RAS (Piersma et al., 2015). Evidence suggests that Ang II can modulate Wnt signaling components, influencing fibroblast differentiation and ECM dynamics (Konigshoff et al., 2008). Targeting this crosstalk, perhaps via agents that simultaneously inhibit RAS and aberrant Wnt signaling, represents a potential multi-target strategy.

Emerging research highlights the role of non-canonical RAS members, such as Ang- (1–7) acting through the Mas receptor, in counteracting fibrotic remodeling. Studies in cardiovascular fibrosis demonstrate that the ACE2/Ang-(1–7)/Mas axis attenuates pathological remodeling by opposing Ang II/AT1R actions, reducing inflammation, and inhibiting key fibrotic pathways including those mediated by TGF-β. () While most evidence originates from cardiac and vascular studies, this protective axis is also present in skin. Its targeted stimulation—via Ang-(1–7) analogs or ACE2 activators—presents a novel therapeutic avenue complementary to AT1R blockade, aiming to rebalance the local RAS towards an antifibrotic state in scars.

Furthermore, the Hippo signaling pathway, a key regulator of organ size and tissue homeostasis via effectors Yes-associated protein/Transcriptional co-activator with PDZ-binding motif (YAP/TAZ), has been implicated in fibrosis (Piersma et al., 2015). Mechanical stress, a known aggravator of scarring, can activate YAP/TAZ, which in turn can synergize with TGF-β signaling (Meng et al., 2016). Preliminary data suggest potential crosstalk between RAS and Hippo pathways in fibrotic contexts, though this remains underexplored in cutaneous scarring. Unraveling these connections could reveal new nodes for intervention, especially in scars subject to high tension.

In conclusion, these interactions position the RAS not as an isolated driver, but as a central hub within a broader fibrotic signaling network. Future therapeutic strategies may benefit from targeting these interconnected pathways, either through multi-target drugs or rational combination therapies.

### 3.3 Interaction with inflammation and oxidative stress

The pathogenesis of pathological scars is not solely fibrotic but also inflammatory in nature. Ang II exerts a profound influence on the inflammatory microenvironment by recruiting and modulation of immune cells (Lucas et al., 2025). AT1R signaling has been demonstrated to promote the infiltration and polarization of macrophages towards the M2 phenotype, which in turn secrete TGF-β1 and platelet-derived growth factor, thereby further exacerbating fibrosis. Furthermore, the process recruits mast cells and lymphocytes, thereby sustaining chronic inflammation (Huang and Ogawa, 2020).

At the molecular level, Ang II-induced activation of NF-κB and signal transducer and activator of transcription 3 (STAT3) reinforces the expression of inflammatory cytokines (e.g., tumor necrosis factor-alpha (TNF-α), interleukin-6 (IL-6), and interleukin-1β (IL-1β)), creating a positive feedback loop between inflammation and fibrosis. Furthermore, Ang II has been demonstrated to stimulate ROS generation, which in turn activates latent TGF-β, thus establishing a direct link between oxidative stress and enhanced profibrotic signaling (Tan et al., 2018; Forrester et al., 2018b). This crosstalk thereby establishes a vicious cycle of persistent inflammation and fibrosis that is central to the progression of scarring.

### 3.4 Dual source of ACE (team’s unique contribution)

**FIGURE 2:**
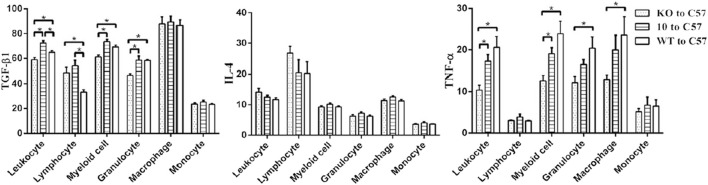
Growth factor (TGF-β1, interleukin-4 (IL-4), and TNF-α) levels in C57 mice that underwent bone marrow transplantation. Wound tissues were harvested in the middle of the wound-healing period (4 d after the operation). Data are means ± SEM. *P < 0.05 (LSD and SNK tests) (Fang et al., 2018a). Copyright © FASEB.

The research of our group has provided novel insights into the origin of ACE during scar formation. Utilizing bone marrow transplantation models and skin tissue analyses, we demonstrated that ACE originates from two distinct sources: bone marrow-derived inflammatory cells (e.g., macrophages) and skin-resident cells (e.g., fibroblasts, keratinocytes). The disruption of either source of ACE has been shown to significantly attenuate scar formation by reducing inflammatory infiltration and suppressing TGF-β1 expression (Fang et al., 2018a). This dual-source model highlights the multifaceted contribution of ACE to local RAS activation in scars and underscores its value as a therapeutic target (Figure 2).

## 4 Targeting the RAS: therapeutic strategies

**FIGURE 3:**
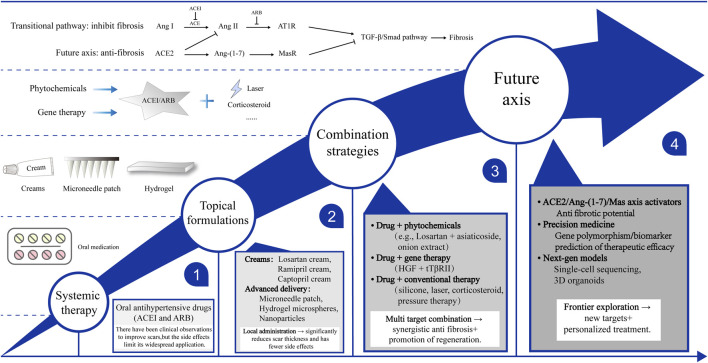
Current and emerging therapeutic strategies targeting RAS for pathological scarring.

It is evident that the RAS plays a pivotal role in the formation of pathological scar tissue. Consequently, intervention strategies that target this system have emerged as a research hotspot in anti-fibrotic therapy. The prevailing approaches in this field primarily concentrate on the inhibition of Ang II production or the prevention of its binding to AT1R. These approaches encompass a variety of levels, including systemic administration, local delivery, combination therapies, and emerging technologies (Table 1). This review systematically examines the mechanistic rationale, efficacy characteristics, and translational potential of existing strategies, while exploring future directions for optimization (Figure 3).

**TABLE 1:** Summary of studies evaluating RAS-targeted agents in scar models and patients in the skin.

Year	Drug/Agent	Administration route	Model (animal/Clinical)	Mechanism of action	Main outcomes	References
Systemic administration						
2006	Enalapril (ACEI, low dose)	Oral	Clinical case reports (postsurgical abdominal keloid scar)	Modulation of systemic RAS	The keloid scar improved, with a nearly complete recovery	Iannello et al. (2006)
2013	Candesartan cilexetil (ARB)	Oral	Case report (a woman aged 63 years with hypertension who had extensive keloids covering her right arm)	—	Improved the objective symptoms of the remaining keloids	Ogawa et al. (2013)
2013	Enalapril (ACEI)	Oral	Rabbit ear wound model	Down-regulatory effects on type III collagen production	Decreased the scar elevation index and fibroblast and capillary counts	Uzun et al. (2013)
2018	Enalapril (ACEI) and candesartan (ARB)	Oral	Rabbit ear hypertrophic scar model	Modulation of fibroblast count and collagen	Reduced scar tissue development	Demir et al. (2018)
2018	ACEIs	Oral	Rat scar model	Suppressed both TGF-β1/Smad2/3 and TGF-β1/TAK1 pathways	Inhibited scar formation	Fang et al. (2018b)
2018	ACEIs	Oral	Mouse scar model (ACE KO male mice)	Inhibition of TGF-β/Smad and TAK1 pathways	Reduced scar thickness and collagen density	Tan et al. (2018)
2019	Losartan	Oral	Rat scar model	Decreases myofibroblast activity and reduces monocyte trafficking	Prevention of hypertrophic scars	Murphy et al. (2019)
2020	ACEIs/ARBs	Oral	Clinical (thyroidectomy patients, retrospective cohort)	Reduced Vancouver Scar Scale scores	Reduced scar formation	Hu et al. (2020)
2020	Valsartan (ARB) and enalapril (ACEI)	Oral	Rabbit ear wound model	Reduction of fibroblast count, capillary count, type 1/3 collagen ratio, collagen organization, and epithelial thickness	Prevention of pathological scar formation	Kurt et al. (2020)
2021	Captopril (ACEI)	Oral	Normotensive rat, the Wistar Kyoto rat (WKY) and the spontaneously hypertensive rat (SHR)	Decreased expression of α-SMA, marker of proliferation Ki-67 (Ki67), and vascular endothelial growth factor (VEGF)	Reduced scars in hypertensive rats than in normotensive rats	Rha et al. (2021)
2023	ACEIs/ARBs	Oral	A retrospective chart review (received bilateral breast reduction surgery over a 10-year period)	—	Reduction in the incidence of hypertrophic scarring	Dow et al. (2023)
2025	Losartan (ARB)	Oral	Normotensives rats, spontaneously hypertensive rats and hyperglycemic rats	Regulating TGF-β/Smad pathway and collagen organization	Reduced skin and collagen fiber thickness	Garcia et al. (2025)
Topical formulations						
1997	Vasoconstrictor peptide angiotensin II (Ang II) +losartan (ARB)	Injection	Rat subcutaneous sponge granuloma model	Inhibition of Ang II-enhanced angiogenesis	Balanced angiogenesis	Walsh et al. (1997)
2008	Captopril (ACEI)	Topical application	Rabbit ear wound model	—	Increased in collagen organization, while decreased in collagen organization scale	Safaee et al. (2008)
2009	Captopril (ACEI)	Topical cream	Clinical case report (patient with a history of a burning accident)	—	Decreased the keloid lesion and eliminated redness and scaling without itchiness and systemic side effects	Ardekani et al. (2009)
2012	ACEI + COX-2 inhibitor	Topical application	Rabbit ear wound model	Celecoxib inhibited the initial inflammation and captopril inhibited scar elevation	Prevention of hypertrophic scarring	Kim et al. (2012)
2016	Enalapril (ACEI)	Injection	Case report (a male aged 30 years with five chest keloids)	—	A 30% improvement in scar softness and a 20% decrease in height	Alexandrescu et al. (2016)
2018	Losartan (ARB)	Topical cream	Randomized controlled trial (patients with hypertrophic scars and keloids)	—	Reduced Vascularity and pliability	Hedayatyanfard et al. (2018)
2019	Ramipril (ACEI) and losartan (ARB)	Topical cream	C57BL/6 mouse scar model	May reduce the TGF-β1	Inhibited scar formation	Zheng et al. (2019)
2022	Losartan (ARB)	Topical cream	Murine full-thickness excision model	Inhibition of TGF-β/Smad signaling	Inhibition of scarring	Zhao et al. (2022)
2023	Losartan (ARB)	Microneedle patch	Rabbit ear hypertrophic scar model	Transdermal delivery of losartan and down-regulating the gene expression of TGF-β1, IL-6 and collagen I	Marked reduction in scar thickness and collagen density	Huang et al. (2023a)
2023	Enalapril (ACEI)	Injection	Forty patients with multiple keloids	—	VSS and POSAS had difference with before treatment, lower complications than TAC	Hamada et al. (2023)
2024	AT2R agonist (C21) and AT2R antagonist (PD123319)	Topical application	Mice and splinted excisional wound model	Decreased inflammatory cell infiltration and pro-inflammatory gene transcription	Near-normal collagen density and collagen I:III ratio, increased vascular density	Harrison et al. (2024)
2025	Alginate hydrogels containing losartan (ARB)	Hydrogel	Rats with a full-thickness model	Decreased in expression level of TGF-β1 and VEGF	Decreased the size of the scar and tissue remodeled	Zamani et al. (2025)
2025	Aloe-emodin and emodin (Chinese herb compounds)	Topical cream	Rabbit ear scar model	Inhibition of AT1R/NF-κB; stimulation of AT2R/MMP-1	Improved scar outcomes; novel multi-target approach	Qu et al. (2025)

### 4.1 Systemic administration (repurposed antihypertensives)

The repurposing of ACEIs and ARBs which are widely used as antihypertensive drugs—has opened new therapeutic perspectives for pathological scarring. The extant clinical evidence suggests that patients receiving long-term oral ACEIs or ARBs exhibit improved scar quality after surgery when compared with those on other antihypertensive regimens. Hu et al. demonstrated in a retrospective cohort of thyroidectomy patients that systemic ACEI/ARB users had significantly lower Vancouver Scar Scale scores, with more organized collagen alignment and reduced inflammatory infiltration (Hu et al., 2020). Similar findings have been reported in reviews emphasizing the antifibrotic effects of these agents across organ systems, reinforcing their translational value for scar management (Maranduca et al., 2023; Greif et al., 2023).

Notwithstanding these encouraging results, systemic administration is inherently limited in its application. Adverse effects such as cough, hyperkalemia, and hypotension restrict their use in normotensive individuals or those with comorbidities.

### 4.2 Topical formulations

In order to circumvent systemic side effects, the topical delivery of ACEIs and ARBs has been the subject of considerable research. The development of losartan and ramipril creams, with optimized penetration enhancers such as chitosan and azone, has been undertaken by our group and others. This development has resulted in a significant improvement in drug retention in the skin. Preclinical studies in murine and rabbit models demonstrated the efficacy of topical formulations in reducing scar hypertrophy. This was achieved by attenuating TGF-β/Smad signaling, while avoiding systemic hypotension (Zhao et al., 2022; Fang et al., 2018b).

Recent innovations have explored advanced delivery systems. For instance, Huang al. developed losartan-loaded microneedle patches that enabled targeted intradermal delivery, resulting in significant reductions in scar thickness and collagen density in hypertrophic scar models (Huang Y. et al., 2023). These advancements suggest that localized RAS modulation is not only effective but also safer, thereby laying the foundation for future clinical translation.

### 4.3 Combination and next-generation strategies

In addition to monotherapy, combination approaches are under active development. Compound topical formulations integrating ARBs with phytochemicals, such as asiaticoside or onion extract, have shown synergistic inhibition of fibrosis by targeting multiple profibrotic pathways (Zhao et al., 2022). Gene therapy strategies are also being investigated; co-delivery of truncated transforming growth factor-beta receptor II (tTβRII) with hepatocyte growth factor (HGF) in animal scar models effectively suppressed fibrosis while promoting epithelial regeneration, illustrating a dual mechanism of antifibrotic and regenerative action (Xu et al., 2020).

Furthermore, RAS-targeted agents may be combined with conventional therapies, such as silicone sheeting, corticosteroid injections, or laser treatments, to enhance efficacy and reduce recurrence rates. Finally, emerging evidence suggests that the ACE2/Ang-(1–7)/Mas receptor axis exerts protective antifibrotic effects, representing a novel therapeutic direction complementary to traditional AT1R blockade (Shenoy et al., 2010). Future strategies may include agents or biologics that enhance this counter-regulatory axis, providing a more balanced modulation of local RAS activity in scar tissue.

### 4.4 Emerging direction: potential of epigenetic regulation in scar treatment

In recent years, beyond classical signaling pathways, epigenetic regulation (such as DNA methylation, histone modifications, and non-coding RNAs) has been demonstrated to play a critical role in fibrotic diseases (Lin et al., 2025; Stratton and McKinsey, 2016; Huang R. et al., 2023). These epigenetic mechanisms dynamically regulate the expression of pro-fibrotic genes, influencing fibroblast activation, inflammatory responses, and ECM remodeling (Jin et al., 2025; Lv et al., 2020; Grafanaki et al., 2025; Gu et al., 2026). Therefore, exploring the interaction between RAS and epigenetic regulation (Gu et al., 2026; Stevenson et al., 2022; Almier et al., 2025) may offer new insights into the persistence and individual variability of scar formation. In the analysis of the methylome and transcriptome data of fibroblasts from normally nourished mature scars and their uninjured counterparts on the contralateral side, Stevenson et al. revealed that Forkhead box F2 (FOXF2) is a key regulator in this process. The targeting of genes responsible for maintaining scar phenotypes may result in improvements to scar appearance and enhanced patient prognosis (Stevenson et al., 2022). The combined application of RAS inhibitors and epigenetic modulators may offer novel approaches for achieving more durable and fundamental anti-fibrotic effects in the future.

Legend: The following schematic overview outlines current and emerging therapeutic approaches that target the RAS in pathological scarring. Repurposed systemic ACEIs/ARBs have been demonstrated to possess potential antifibrotic effects; however, limitations are imposed by systemic side effects. The utilization of topical formulations, encompassing creams, microneedle patches, and hydrogels, facilitates localized delivery, thereby enhancing safety. Combination strategies involve the integration of RAS inhibitors with phytochemicals, gene therapy, or conventional therapies with a view to enhance efficacy. Future directions in this field emphasize the ACE2/Ang-(1–7)/Mas axis and next-generation precision medicine approaches.

## 5 Limitation of current evidence and future perspectives

Despite mounting evidence supporting the RAS as a promising target for pathological scarring, its translation into routine clinical practice faces significant hurdles. This section outlines the key challenges, critically appraises the limitations of existing evidence, and proposes future directions (Table 2).

**TABLE 2:** Translational limitations, challenges, and future perspectives in RAS-targeted scar therapy.

Challenge	Description	Potential solution/Future direction	References
Lack of large-scale and reliable RCTs	Current evidence mainly from animal studies or small cohorts and the risk of bias was high	Conduct multicenter, randomized controlled trials, especially on topical ACEI/ARB formulations or applying drugs immediately after skin injury	Greif et al. (2023)
Individual variability (e.g., ethnicity)	Genetic background influences responsiveness to RAS-targeted drugs	Incorporate pharmacogenomics and biomarker discovery for personalized treatment	Du et al. (2024)
Safety concerns with systemic therapy	Hypotension, cough, hyperkalemia limit broad application	Prioritize localized/topical formulations with minimal systemic exposure	Hu et al. (2020)
Drug delivery limitations	Poor penetration and short half-life in conventional creams	Develop advanced systems (microneedles, nanoparticles, hydrogels) to enhance local delivery	Huang et al. (2023a)
Limited mechanistic resolution	Scar heterogeneity across fibroblast and immune subtypes remains unclear	Apply single-cell RNA sequencing and 3D organoid models to map RAS heterogeneity	Shim et al. (2022)

### 5.1 Limitations of current evidence

#### 5.1.1 Lack of high-quality clinical evidence

Clinically, the evidence is predominantly observational (retrospective cohort studies, case reports, and series) or consists of small, often non-randomized trials. There is a conspicuous lack of large-scale, multicenter, RCTs with robust blinding, standardized outcome measures (e.g., Patient and Observer Scar Assessment Scale (POSAS), Vancouver Scar Scale (VSS), histology), and long-term follow-up to assess recurrence (Greif et al., 2023; Hu et al., 2020).

Moreover, the majority of clinical trials necessitate only the approval of local medical institutions or ethics committees, making it difficult for researchers to access the latest clinical trial results and resulting in researchers' insufficient comprehension of the efficacy of RAS-targeted therapy in humans. Consequently, the number of studies included in this review is limited, and the focus is primarily on completed and published research (Table 3). This evidence gap makes it difficult to establish definitive treatment protocols, optimal dosing (for both prevention and treatment), and timing of intervention.

**TABLE 3:** The ongoing and completed clinical trials investigating in RAS-targeted scar management.

Registration ID	Primary objective & design	Intervention	Primary endpoint & key results	Safety profile	Status and publication
Two cases (No ethical number provided)	The fortuitous observation of a beneficial effect of enalapril on recent and long-standing postsurgical keloid scars	Low dose of enalapril orally	Postsurgical abdominal keloid scar	Not provided	Completed and published (Iannello et al., 2006)
Report of the first case. Written informed consent was obtained from patient	This case represents the first human case successfully treated with topical captopril as a new anti-keloid agent	5% captopril cream	6 weeks of twice-daily application of the 5% captopril cream	No itchiness and without any cutaneous or systemic side effects	Completed and published (Ardekani et al., 2009)
Case report (No ethical number provided)	A 63-year-old woman had hypertension together with severe keloids that covered her right elbow, wrist joints, and thumb and made it difficult for her to use her right hand	The contractures were released by surgery and postoperative radiation therapy. The internal medicine clinic started her on a Ca-channel blocker and an angiotensin II blocker	Not provided	Not provided	Completed and published (Ogawa et al., 2013)
Ethics Committee at Shahid Beheshti University of Medical Sciences, Tehran (SBMU.REC.1392.198)	A single-blind and randomized controlled trial to evaluate the clinical effects of losartan ointment on reducing hypertrophic scar and keloid	Losartan 5% and placebo treatment groups	The treatment was performed twice a day for 3 months and a 6-month follow up	No allergy and hypotensive signs	Completed and published (Hedayatyanfard et al., 2018)
Ethics Committee of Shiraz University of Medical Sciences (No number provided)	A single-center, double-blind, prospective clinical trial to evaluate the efficacy of enalapril in reduction of hypertrophic scar size and itching	1% enalapril and placebo treatment groups	One side was treated with 1% enalapril ointment and the other side with placebo and twice daily for a period of 6 months	With low incidence of adverse drug reactions	Completed and published (Mohammadi et al., 2018)
Ethics committee of the Fourth Affiliated Hospital Zhejiang University School of Medicine (Ethical number: 201,503,172)	A questionnaire-based observational study aims to investigate whether ACEI and ARB could inhibit scar formation in humans	ACEI group, ARB group, other antihypertensive drugs control group and blank control group	At least 5 months between the thyroidectomy and our investigation, taking antihypertensive drugs all the time after operation	Not provided	Completed and published (Fang et al., 2018b)
Ethics approval was obtained from the local Research Ethics Board, file number: # 1023867	A retrospective chart review to assess whether the use of ACEI or ARBs undergoing reduction mammoplasty is correlated with a reduction in hypertrophic scarring post-operatively	Treated with either an ACEI or ARB	Not provided	Not provided	Completed and published (Dow et al., 2023)
Institutional Review Board (IRB) of the Faculty of Medicine, Fayoum University, IRB 00003613	A prospective randomized comparative study to evaluate the effectiveness of intralesional injection of enalapril versus triamcinolone acetonide in keloids	Enalapril injection and triamcinolone acetonide injection	Injected intralesional in one session per month for three sessions. Examined by two experienced dermatologists before and 3 months after the last treatment	Regarding adverse effects, 35% of patients had no symptoms, 29% of patients complained of pain, 30% complained of itching, and 6% complained of tenderness	Completed and published (Hamada et al., 2023)
NCT05259137	A randomized, double-blind, human clinical trial with a paired split-scar design to test the use of enalaprilat for intralesional injection in a hypertrophic scar	1 mL of 10 mg/mL triamcinolone acetonide and 1 mL of 10 mg/mL triamcinolone acetonide +1.0 mL of 1.25 mg/mL of enalaprilat	Injection at 0 weeks, 6 weeks, and 12 weeks	To mitigate risk in the unexpected event of an allergic and/or anaphylactic reaction, participants will be briefly monitored after enalaprilat injection	Withdrawn
NCT05893108	A randomized, double-blind, clinical trial to compare the efficacy of pre and post-treatment and between 5% losartan in ethosomal gel and 10 mg/mL triamcinolone acetonide injection	Ethosomal gel bearing losartan 5% and triamcinolone acetonide 10 mg/mL	Ethosomal gel bearing losartan 5% applied two times a day for three consecutive months on keloids; Intralesional injection of triamcinolone acetonide 10 mg/mL every 2 weeks for three consecutive months on keloid	Not provided	Unknown

#### 5.1.2 Heterogeneity and lack of patient stratification

Compared to human scarring, most experimentally designed animal models fail to fully replicate the complexity of human scar pathophysiology, particularly the chronicity and heterogeneity of keloids (Greif et al., 2023; Hu et al., 2020). Concurrently, significant individual variation exists in scar susceptibility and potential response to RAS-targeted therapies. Genetic polymorphisms in ACE (e.g., I/D polymorphism), ATR1, and other RAS components can influence local RAS activity and drug responsiveness (Du et al., 2024). The lack of validated predictive biomarkers makes it impossible to identify which patients are most likely to benefit, leading to potential trial-and-error approaches and suboptimal outcomes. Furthermore, the differential effects of various ACEIs and ARBs in scarring are poorly understood, as most studies lump these classes together or focus on single agents (e.g., losartan, enalapril).

#### 5.1.3 Safety concerns associated with systemic administration

While ACEIs/ARBs have an excellent safety profile for hypertension, their repurposing for a non-hypertensive indication like scarring raises different concerns. Systemic administration, even at lower doses, can cause hypotension, hyperkalemia, or cough, limiting its use in normotensive individuals, especially the young—a key demographic for scar prevention (Maranduca et al., 2023; Hu et al., 2020).

#### 5.1.4 Drug delivery hurdles for topical applications

Topical formulations have been demonstrated to mitigate systemic risks; however, the effective delivery of drugs to the deep dermal fibroblast bed remains a challenging area of research. The stratum corneum is a formidable barrier, and scar tissue itself has altered vascularity and dense ECM, impeding penetration. Conventional creams have been shown to exhibit suboptimal bioavailability and brief residence time in the skin (Huang Y. et al., 2023; Zhao et al., 2022).

#### 5.1.5 Incomplete mechanistic resolution

The complexity of RAS crosstalk with other key pathways (e.g., Wnt, Hippo, epigenetic regulators) in the context of scarring is not fully elucidated. The heterogeneity of fibroblast and immune cell subtypes expressing RAS components in scar tissue remains largely unexplored at a single-cell level.

### 5.2 Future perspectives: targeted solutions for current limitations

#### 5.2.1 Prioritizing high-quality clinical research

To overcome the evidence gap, well-designed, large-scale RCTs are paramount. Future trials should evaluate standardized (preferably topical) RAS inhibitor formulations against placebo or standard care in clearly defined patient populations (e.g., post-surgical, high-risk individuals). Long-term studies are needed to assess durability of effect and recurrence rates.

#### 5.2.2 Embracing precision medicine approaches

To address patient heterogeneity, pharmacogenomics and biomarker discovery must be integrated into future trial designs. Stratifying patients by ACE genotype or baseline levels of local RAS components could enable personalized therapy, maximizing efficacy for responders and avoiding unnecessary exposure for non-responders (Du et al., 2024).

#### 5.2.3 Optimizing localized delivery

Given the safety limits of systemic use, the field must prioritize optimized topical and transdermal delivery systems. Advanced technologies such as microneedle arrays (Huang Y. et al., 2023), nanoparticle carriers (liposomes, polymeric nanoparticles), and injectable hydrogels (Yao et al., 2026) can bypass the stratum corneum, enhance local drug concentration, and provide sustained release, maximizing efficacy while ensuring safety.

#### 5.2.4 Advanced mechanistic and technological integration

To deepen mechanistic resolution, future research should employ cutting-edge technologies. Firstly, single-cell RNA sequencing and spatial transcriptomics can map RAS component expression across cellular subtypes in human scar samples, identifying novel cell-specific targets (Shim et al., 2022). Furthermore, exploration of the protective ACE2/Ang-(1–7)/Mas axis (via agonists or gene therapy) represents a promising strategy to rebalance dysregulated local RAS (Zhong et al., 2025; Shenoy et al., 2010). Investigating epigenetic regulators (e.g., microRNAs (Lee et al., 2022), histone modifiers) that interact with RAS signaling could uncover new nodes for intervention. Future research should explore combining these epigenetic editing tools with RAS-targeted therapies to achieve more durable anti-fibrotic effects (Tu et al., 2025).

#### 5.2.5 Rational combination strategies

To enhance efficacy and reduce recurrence, future studies should explore rational combination therapies. This could involve combining RAS-targeted agents with conventional therapies (e.g., corticosteroids, silicone sheeting, laser therapy) or with agents targeting complementary fibrotic pathways (e.g., Wnt inhibitors, pro-regenerative factors) identified through mechanistic studies.

In conclusion, while the pathophysiological rationale for targeting RAS in scarring is strong, the translational journey requires concerted efforts. Future success hinges on generating robust clinical evidence, developing sophisticated delivery platforms inspired by interdisciplinary advances, and embracing a precision medicine framework to transform this promising mechanistic insight into tangible clinical benefits for patients.

## 6 Conclusion

The RAS has been identified as a pivotal catalyst for the development of pathological scarring, with its functions extending significantly beyond its conventional role in blood pressure regulation. Increasing evidence indicates that Ang II/AT1R signaling orchestrates a network of profibrotic and proinflammatory pathways, including TGF-β/Smad, TAK1/NF-κB, and MAPK cascades, and intersects with other key pathways like Akt and Wnt/β-catenin, sustaining extracellular matrix accumulation, chronic inflammation, and scar hypertrophy (Maranduca et al., 2023; Greif et al., 2023; Mehta and Griendling, 2007; Konigshoff et al., 2008).

From a therapeutic standpoint, RAS inhibition through ACEIs and ARBs has been demonstrated to be a highly effective strategy in the mitigation of scar formation. As demonstrated by preclinical and early clinical studies, both systemic and topical applications have shown antifibrotic efficacy. Topical formulations, in particular, offer targeted local delivery while minimizing systemic side effects, representing a promising avenue for translation into clinical practice (Zhao et al., 2022; Hu et al., 2020). Recent advances such as microneedle-mediated delivery, nanoparticle systems, and biomaterial-based strategies (e.g., hydrogels) inspired by regenerative medicine approaches further enhance the feasibility and potential efficacy of localized RAS-targeted therapy (Huang Y. et al., 2023; Zhong et al., 2025).

From mechanistic discovery to translational application, the research of our group has provided fundamental evidence for the role of ACE in scar biology. This includes the finding that ACE originates from both bone marrow–derived and skin-resident cells, and the ability of ACEI/ARB to simultaneously suppress canonical and noncanonical TGF-β pathways (Fang et al., 2018a; Fang et al., 2018b). These insights, combined with the growing understanding of RAS crosstalk with other pathways and the protective ACE2/Ang-(1–7)/Mas axis, establish RAS modulation as a rational, mechanistically grounded strategy for scar prevention and treatment.

In the future, overcoming the current limitations—through high-quality RCTs, precision medicine based on biomarkers and genetics, and advanced delivery technologies—will be crucial. Furthermore, the targeting of epigenetic regulatory mechanisms has been demonstrated to have unique potential for combating fibrosis. A combinatorial approach, integrating RAS-targeted therapy with conventional interventions (e.g., silicone sheets, corticosteroids, laser therapy) or with agents targeting complementary pathways, may optimize therapeutic outcomes and reduce recurrence. The integration of innovative drug delivery systems, multi-targeted regimens, and advanced mechanistic tools such as single-cell sequencing and organoid models will be essential for the advancement of this field. These converging innovations suggest that RAS-targeted therapies have the potential to transform the landscape of scar management and provide new hope for patients suffering from pathological scarring (Maranduca et al., 2023; Huang Y. et al., 2023; Shim et al., 2022).
